# Finally finished! National Competence Based Catalogues of Learning Objectives for Undergraduate Medical Education (NKLM) and Dental Education (NKLZ) ready for trial

**DOI:** 10.3205/zma000977

**Published:** 2015-08-17

**Authors:** Martin R. Fischer, Daniel Bauer, Karin Mohn, NKLM-Projektgruppe

**Affiliations:** 1 Gesellschaft für Medizinische Ausbildung (GMA), Erlangen, Deutschland; 2 Klinikum der Universität München, Institut für Didaktik und Ausbildungsforschung in der Medizin, München, Deutschland; 3 Universität Osnabrück, Fachbereich Humanwissenschaften, Osnabrück, Deutschland

## Note

Finally finished! Earlier this June, the National Competence Based Catalogues of Learning Objectives for Undergraduate Medical Education (NKLM) and Dental Education (NKLZ) were passed with overwhelming agreement at the annual meeting of the Association of Medical Faculties in Germany (MFT) [http://www.mft-online.de/files/pm_nklm-nklz_2015-07-27.pdf, cited 2015-08-03] after nearly six years of development. The catalogues describe the competencies students of medical or dental degrees in Germany should have acquired by the time they graduate. They were made freely available online not long ago at www.nklm.de (www.nklz.de respectively).

What were the starting points for the development process? On the one hand the institute tasked with the execution of the written licensing examinations (Institut für Medizinische und Pharmazeutische Prüfungsfragen, IMPP) has long provided catalogues which list topics to be assessed via multiple choice tests. But much remained unclear. They list diseases and leading symptoms relevant for the clinical subjects but without elucidating what knowledge and which abilities a newly licensed physician should have exactly – i.e. listing diabetes without mentioning interdisciplinary tasks in secondary prevention, communication with patients and relatives, and collaboration with diabetes educators. The NKLM does not order its content by subject or organ; subjects associated with an item are considered suggestions, leaving the mapping of competencies and local departments responsible for their mediation to the individual faculties.

To what extent should doctor-patient communication be mastered and in which contexts? Working in professional teams? Skills of scholarship and critical appraisal of studies? Experience in conducting research including literature searches, developing research questions and hypotheses? All this content and competencies indeed had already been identified by faculties and were being conveyed to a certain extent.

Many countries already provide competence-based descriptions of medical programs - most notably Canada, the Netherlands and Switzerland. These served as reference and a basis for discussion in the development of NKLM.

In 2009, the Society for Medical Education (Gesellschaft für Medizinische Ausbildung, GMA) and the Association of Medical Faculties in Germany (Medizinischer Fakultätentag, MFT) were commissioned by the Standing Conference of Ministers of Education and Cultural Affairs’s Higher Education Committee (Hochschulausschuss der Kultusministerkonferenz der Länder) to develop a competence-based catalogue of learning objectives for medicine to aid faculties in the advancement and modernization of their curricula [1]. The NKLM was to define the foundations for students moving on to postgraduate clinical education. The road to completion was longer and more complicated than originally anticipated though.

21 teams incorporating over 200 medical experts worked out a draft, then presented to a steering committee that included all major stakeholders in medical education and postgraduate training, in particular the German Medical Association (Bundesärztekammer), the National Association of Medical Students in Germany (Bundesvereinigung der Medizinstudierenden Deutschlands, bvmd), and the Association of the Scientific Medical Societies in Germany (Arbeitsgemeinschaft der Wissenschaftlichen medizinischen Fachgesellschaften, AWMF). The draft was discussed and a version agreed upon which then underwent systematic revision by all scientific medical societies of the AWMF (over 160) via an online platform.

Over two years, 98 societies participated in a two-step Delphi consensus process continuously improving and commenting the drafts (with over 4000 comments submitted in the first Delphi round alone). With success. Eventually, all 234 competencies and 281 sub-competencies reached strong agreement or consensus and were included in the catalogue’s final version. Just two of the proposed 1958 learning objectives that further operationalize and describe the competencies could not be agreed upon. The NKLM office at the GMA coordinated this process (with the kind support of the Robert Bosch Foundation) and compiled the catalogue’s final version in close cooperation with the MFT.

Competencies and sub-competencies listed in the catalogues in their present form have the status of recommendations for medical faculties while the library of learning objectives provided will have to be trialed. It can be assumed that with time the catalogue’s volume will decrease – based on the Swiss experience, where the Swiss Catalogue of Learning Objectives’ content was reduced by 30% from the first to the revised second edition [http://sclo.smifk.ch/, cited 2015-08-04].

From IT infrastructure to concept design for competence-based assessment, challenges remain and will have to be faced. Time will show what kind of support faculties and individual teachers will need in the implementation of the NKLM. An expert group has been set up by the MFT to support this implementation process. Until 2020 the faculties’ experiences with the catalogues are to be regularly exchanged and discussed, and will give insights into what future revisions and developments should address.

It will be interesting to see to what extent medical education in Germany will turn competence-based and what impact this will have on the federal government’s Master Plan 2020 on Medical Education (Masterplan Medizinstudium 2020). Published in 2014, the Science Council (Wissenschaftsrat)’s much-discussed paper on the advancement of medical education in Germany, based on experiences from reformed medical programs, prominently and repeatedly points at the NKLM as an important basis for future medical education in Germany [http://www.wissenschaftsrat.de/download/archiv/4017-14.pdf, cited 2015-08-04].

The path to completion of NKLM and NKLZ was tedious and accompanied by controversy over the desirability and feasibility of competency-based medical education. Indeed the catalogues’ implementation and real-life trials at the medical faculties will not be less arduous but point the way to the future of medical education in Germany. It is about nothing less than ensuring future doctors are trained as well as humanly possible to face the health system’s challenges and to adequately carry out their various professional roles for the benefit of their patients. Many thanks to all who contributed to the critical discussion and development of NKLM and NKLZ!

## Competing interests

The authors declare that they have no competing interests.
